# Comment on: “A Systematic Review of Organic Versus Conventional Food Consumption: Is There a Measurable Benefit on Human Health? Nutrients 2020, 12, 7”

**DOI:** 10.3390/nu12030696

**Published:** 2020-03-05

**Authors:** Laura Di Renzo, Antonino De Lorenzo, Giuseppe Merra, Paola Gualtieri

**Affiliations:** Section of Clinical Nutrition and Nutrigenomic, Department of Biomedicine and Prevention, University of Tor Vergata, 00133 Rome, Italy; laura.di.renzo@uniroma2.it (L.D.R.); delorenzo@uniroma2.it (A.D.L.); giuseppe.merra@uniroma2.it (G.M.)

**Dear Editor**

We read carefully the review of Vigar V. et al. [1]. Chronic degenerative non-communicable diseases (CDNCD) are the most frequent causes of prolonged disability and death worldwide [2]. Epidemiological studies have shown the associations among diet, lifestyle, and the incidence and severity of CDNCDs. Personalized food patterns have started to be proposed, worldwide, for their prevention [3].

Numerous studies indicate that a healthy and organic diet provides higher content of bioactive compounds and lower content of unhealthy substances, such as persistent pollutants, their metabolites, pesticides, fertilizers [4,5]. Therefore, the consumption of organic food contributes to maintaining an optimal health status and decreases the risk of CDNCD. For these reasons, it is fundamental to the passage from the Environmental to the Health Impact Assessment, in maintaining the nutritional quality of specific food or products along the entire supply chain, according to the Nutrient and Hazard Analysis of Critical Control Point (NACCP) process [6].

A bias highlighted by Vigar V. et al. [1] on the clinical trial data reported was related to the fact that these trials “were not randomized, providing one diet followed by the alternate diet for all participants concurrently”. The authors cited two papers of the same group [7,8]. Actually, in these articles, it is pointed out that the sequential administration of the conventional diet before and the organic one after did not negatively influence the statistical results since a marked improvement in biochemical and body composition parameters was observed only at the end of the last dietetic treatment with organic foods.

Therefore, Vigar V. et al. [1] highlighted the importance of organic food certification, necessary in order to know the true effects on human health. The authors, however, underlined that some papers did not report the certification of organic food used [7,8,9].

About this, it is necessary to dwell on the definition itself, foreseen by the legislation of many countries [10]. In Europe, for example, organic production is regulated by EU Regulation 834/2007. Consumers are guaranteed that when they buy an organic product with an EU label, it is organic. So, Vigar V. et al. [1], in questioning the trustworthiness of results on organic food consumption, make the mistake of underestimating that, at least in Europe, the definition of organic food is dictated by a specific law. For this reason, it is not necessary to define the certification of organic products, as only products that are in accord with the European regulation can be defined as organic and receive the guarantee mark. In any case, De Lorenzo et al. [8], in the introduction of the paper, clearly referred to the European Community Regulations.

Based on these observations, the authors should have been less harsh in evaluating the bias and better considered the works cited, without contesting the observational data.
